# New and distinct chronic wasting disease strains associated with cervid polymorphism at codon 116 of the *Prnp* gene

**DOI:** 10.1371/journal.ppat.1009795

**Published:** 2021-07-26

**Authors:** Samia Hannaoui, Elizabeth Triscott, Camilo Duque Velásquez, Sheng Chun Chang, Maria Immaculata Arifin, Irina Zemlyankina, Xinli Tang, Trent Bollinger, Holger Wille, Debbie McKenzie, Sabine Gilch

**Affiliations:** 1 Department of Comparative Biology and Experimental Medicine, Faculty of Veterinary Medicine; Hotchkiss Brain Institute; University of Calgary, Calgary, Canada; 2 Department of Biological Sciences, Center for Prions and Protein Folding Diseases, University of Alberta, Edmonton, Canada; 3 Department of Biochemistry, Center for Prions and Protein Folding Diseases, University of Alberta, Edmonton, Canada; 4 Western College of Veterinary Medicine, University of Saskatchewan, Canadian Wildlife Health Cooperative (CWHC), Saskatoon, Saskatchewan, Canada; Istituto Superiore di Sanità, ITALY

## Abstract

Chronic wasting disease (CWD) is a prion disease affecting cervids. Polymorphisms in the prion protein gene can result in extended survival of CWD-infected animals. However, the impact of polymorphisms on cellular prion protein (PrP^C^) and prion properties is less understood. Previously, we characterized the effects of a polymorphism at codon 116 (A>G) of the white-tailed deer (WTD) prion protein and determined that it destabilizes PrP^C^ structure. Comparing CWD isolates from WTD expressing homozygous wild-type (116AA) or heterozygous (116AG) PrP, we found that 116AG-prions were conformationally less stable, more sensitive to proteases, with lower seeding activity in cell-free conversion and reduced infectivity. Here, we aimed to understand CWD strain emergence and adaptation. We show that the WTD-116AG isolate contains two different prion strains, distinguished by their host range, biochemical properties, and pathogenesis from WTD-116AA prions (Wisc-1). Serial passages of WTD-116AG prions in tg(CerPrP)1536^+/+^ mice overexpressing wild-type deer-PrP^C^ revealed two populations of mice with short and long incubation periods, respectively, and remarkably prolonged clinical phase upon inoculation with WTD-116AG prions. Inoculation of serially diluted brain homogenates confirmed the presence of two strains in the 116AG isolate with distinct pathology in the brain. Interestingly, deglycosylation revealed proteinase K-resistant fragments with different electrophoretic mobility in both tg(CerPrP)1536^+/+^ mice and Syrian golden hamsters infected with WTD-116AG. Infection of tg60 mice expressing deer S96-PrP with 116AG, but not Wisc-1 prions induced clinical disease. On the contrary, bank voles resisted 116AG prions, but not Wisc-1 infection. Our data indicate that two strains co-existed in the WTD-116AG isolate, expanding the variety of CWD prion strains. We argue that the 116AG isolate does not contain Wisc-1 prions, indicating that the presence of 116G-PrP^C^ diverted 116A-PrP^C^ from adopting a Wisc-1 structure. This can have important implications for their possible distinct capacities to cross species barriers into both cervids and non-cervids.

## Author summary

Chronic wasting disease belongs to the family of prion diseases. It is considered the most contagious prion disease and the only one that affects free ranging wildlife. The disease range is expanding in North America and Northern Europe. This work describes the emergence and characterization of new chronic wasting disease strains related to a polymorphism in the prion protein gene. It supports the concept that strains are a dynamic mixture of substrains that can influence and interfere with each other. Because transmission barriers are governed by the compatibility of a particular prion strain with the new host’s prion protein, it is critical to understand the emergence and variety of chronic wasting disease strains circulating in wild animals and their ability to infect new host species including humans.

## Introduction

Prion diseases or transmissible spongiform encephalopathies (TSEs) are fatal and infectious neurodegenerative disorders. TSEs include Creutzfeldt-Jakob disease (CJD) in humans [1], bovine spongiform encephalopathy (BSE) in cattle, scrapie in sheep and goat and chronic wasting disease (CWD) in cervids [2].

According to the “protein only” hypothesis and structural studies of the pathogenic agent, prions are solely constituted of PrP^Sc^ [3,4], the misfolded isoform of the host-encoded prion protein, PrP^C^. Despite the absence of a nucleic acid genome, experimental studies have revealed the existence of multiple prion strains that exhibit specific phenotypic traits in a given host species that are transmissible upon passages [1,5]. Properties that differentiate prion strains include incubation time, neuropathological features, PrP^Sc^ biochemical properties, host range and tissue tropism [6,7]. All evidence suggests that PrP^Sc^ conformation is responsible for the prion strain diversity, especially the tertiary and/or quaternary structures [8].

CWD is an emerging prion disease that affects deer, elk, reindeer and moose—all members of the cervid family. It was first described and recognized as a TSE in captive mule deer [9], but shortly after, it was also discovered in various cervid species and free-ranging cervids. To date, CWD is the only prion disease of wild animals. The geographic range of CWD continues to expand in North America, recognized, to date, in 26 U.S. states and 3 Canadian provinces. CWD has expanded to new geographic areas, such as South Korea, and Northern Europe, including Norway, Finland and Sweden [10], in a wider range of cervid host species. CWD is considered the most contagious prion disease with horizontal transmission favored by animal interactions and persistence of prion infectivity in the environment [11–13], leading to concerns about the spread of CWD to new cervid and non-cervid species. Because the strain responsible for the emergence of BSE was also the causative agent of new variant CJD in humans [14,15], the possibility of zoonotic transmission of CWD is concerning. Consequently, strain characterization of CWD isolates is essential to predict the fate of emergent strains and their zoonotic potential.

Numerous studies have now demonstrated that there are several CWD prion strains [16–21]. The first evidence of CWD prion strain diversity emerged after transmission of North American CWD isolates of deer and elk into cervid PrP^C^-expressing transgenic mice [22,23]. Angers et al. (23), upon inoculating a panel of CWD isolates from various species and geographic locations into transgenic mice overexpressing deer or elk PrP^C^, identified two CWD prion strains, CWD1 and CWD2, which exist either as strain mixtures or as independent strains. Although CWD1 and CWD2 in transgenic mouse models presented with differences in incubation time and neuropathological profiles, their biochemical properties (electrophoretic mobility, conformational stability, ratio of glycoforms) were indistinguishable [24]. This work shed light on the impact of PrP primary structure on CWD prion strain transmission and their stability. Experimental transmission of PrP wild type-deer prions (Wisc-1) into white-tailed deer (WTD) harboring four different *Prnp* genotypes, with variations at position 95 and 96, resulted in the identification of two strains, the initial Wisc-1 strain and an emerging strain, H95^+^ [17,25]. Previously, we have characterized the impact of a polymorphism at codon 116 (A>G) of the WTD PrP on PrP^C^ and CWD prion properties [26]. Even though the allele encoding this polymorphism at residue 116 (human PrP: 113) is present at only a very low frequency (allelic frequency: 0.13) in the wild population, and its influence on CWD susceptibility remains unclear [27], we became intrigued because it resides in the highly conserved central hydrophobic core domain of PrP, which has implications in prion conversion [28–31].

Compared to Wisc-1 prions, we found that PrP^Sc^ from the 116AG isolate was conformationally less stable, with lower seeding activity in real-time quaking-induced conversion (RT-QuIC) and reduced infectivity *in vitro*. *In vivo*, we serially passaged Wisc-1 and 116AG prions in tg(CerPrP132M)1536^+/+^ mice (referred to as tg1536), overexpressing deer wt-PrP^C^ six to eight-fold [22]. Upon the first passage, mice inoculated with 116AG prions had a longer survival period than mice inoculated with Wisc-1 prions [26]. The disease progression was different, with Wisc-1 inoculated mice having a very rapid disease progression of one week from first symptoms to terminal stage of disease, while mice inoculated with 116AG prions had a very slow disease progression of approximately 3 months [26]. Upon secondary passage in tg1536 mice, the survival period of 116AG-prions was significantly reduced in 80% of the mice [26]. Yet, the conformational features observed in the deer isolates were retained [26]. This finding was a hint at the existence of a new prion strain in the 116AG prion isolate, yet to be proven.

Based on the results of 116AG transmission in tg1536 mice, the goal of the current study was to assess the transmission of WTD isolates in different animal models, specifically to first assert and then identify strains associated with the 116AG isolate. Therefore, we assessed the consequences of prion heterotypic transmission of Wisc-1 and 116AG isolates to wild-type and transgenic rodents expressing different *Prnp* genes, such as wt- and S96-deer PrP, bank voles, and hamsters (S1 Fig).

Our findings contribute to the CWD strain diversity by unveiling the presence of novel strain(s) within the 116AG isolate. In addition, we revealed the presence of two divergent prion strains associated with the WTD-116AG isolate, and the fact that none of them were similar to Wisc-1 strain demonstrates that the presence of the non-wild type116G-PrP^Sc^ may strongly impact the replication of the wt/Wisc-1 PrP^Sc^. Novel strains with such specific characteristics might impact the ability of CWD to cross species barriers among cervid and non-cervid species.

## Results

Prion strains adapt when they are transmitted into the same host species expressing different levels of PrP^C^ molecules [32,33], or are transferred into different host species [34,35]. To understand and compare the transmission and strain properties of CWD isolates from WTD expressing wt PrP and harboring the Wisc-1 strain, or WTD heterozygous for a polymorphism at position 116 (A116G) of the *Prnp* gene, we performed parallel studies in two different laboratories. We assessed the susceptibility to Wisc-1 (WTD-wt) and 116AG prions in four different CWD animal models: two distinct lines of cervid-PrP transgenic mice, bank voles and Syrian golden hamsters. For each transmission, at least five mice, six voles and eight Syrian golden hamsters were inoculated intracerebrally with WTD brain homogenates. The transmission efficiency was evaluated by the appearance and progression of clinical signs of prion disease, assessing the presence of PK-resistant PrP^Sc^ (PrP^res^) by Western blot, as well as observing neuropathological changes in the brain and detecting the morphology of PrP^Sc^ deposits by immunohistochemistry (IHC).

### Serial transmission of WTD prions into tg1536 mice indicated the co-existence of two substrains within the 116AG prions

**Fig 1:**
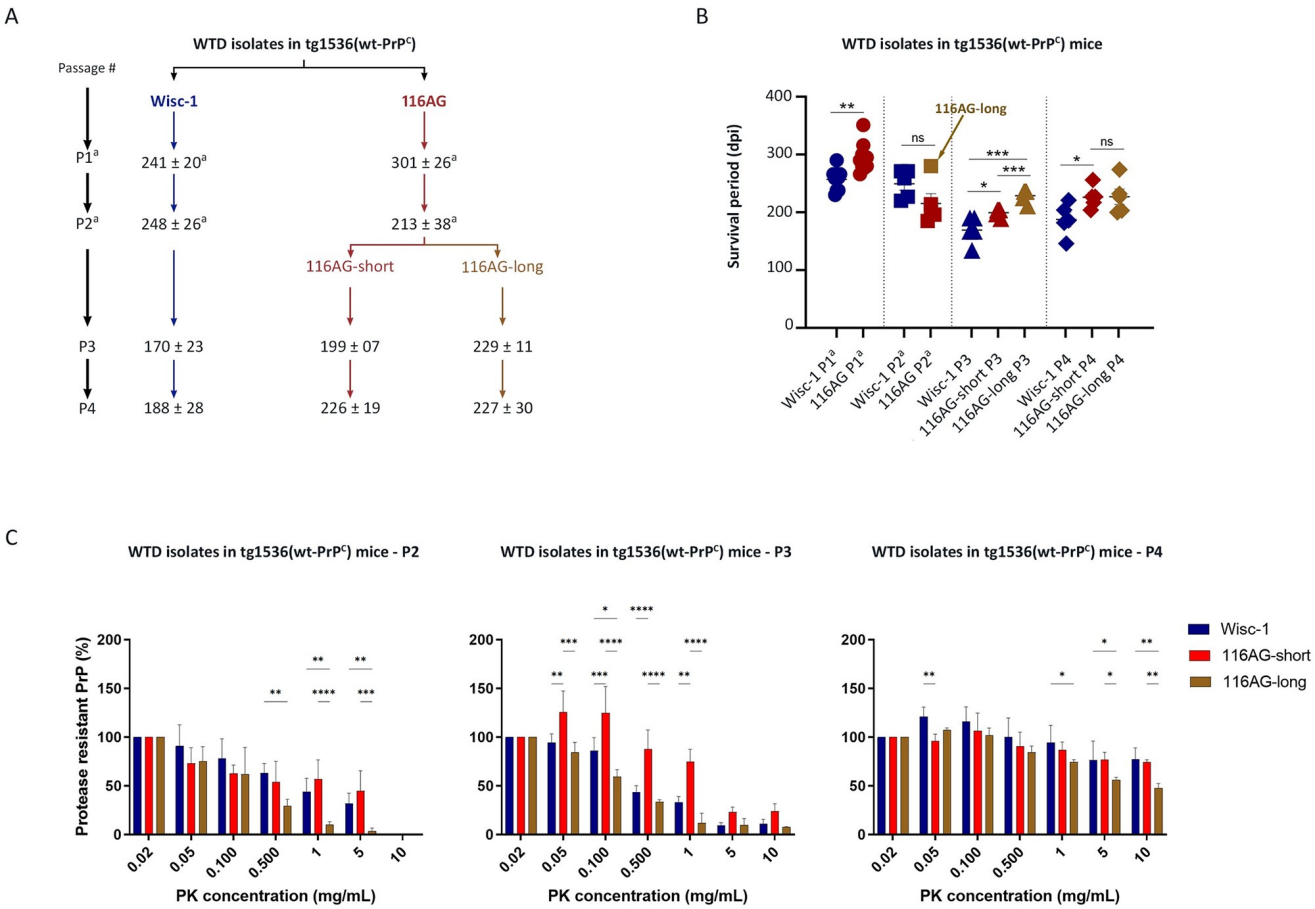
Serial transmission of Wisc-1 and 116AG isolates in tg1536^+/+^ mice. Tg1536^+/+^ mice, overexpressing wt deer PrP 6 to 8fold, were inoculated intracerebrally with 20ul of 1% (w/v) brain homogenate. (A) Scheme of the serial transmission (1^st^ ➔ 4^th^ passage) of Wisc-1 and 116AG isolates to tg1536^+/+^ mice, mean survival times with standard deviation are shown. (B) Representative graph of the survival times of tg1536^+/+^ mice inoculated with Wisc-1 or 116AG prions. The y-axis represents the survival period (dpi) and the x-axis the different passages. Wisc-1 inoculated mice are represented by blue shapes and 116AG inoculated mice are represented by red shapes (filled for 116AG-short and open for 116AG-long). *p < 0.05, **p < 0.01, ***p < 0.001, and ns p > 0.05; unpaired student’s t-test was used to compare between different groups of mice. (C) Resistance to PK was assessed using different concentrations of PK to digest brain homogenates of WTD passaged isolates in tg1536 mice (P2, P3 and P4 represent second, third and fourth passage). The PrP^res^ signal was quantified by densitometric and blotted into graphs using GraphPad Prism software for analyses. *P<0.05, **P<0.01, ***P<0.001 and ****P<0.0001 refers to differences between PK resistance of Wisc-1 (blue bars), 116AG-short (red bars) and 116AG-long (brown bars). Statistical analyses were performed using two-way ANOVA followed by post hoc (Tukey’s multiple comparisons test; GraphPad Prism software). ^a^ data from Hannaoui et al. [26].

Second passage of 116AG prions in tg1536 mice allowed us to distinguish two populations of mice, one with a short survival period (116AG-short) and the second with a longer survival period (116AG-long; [26]; Figs 1 and S2A and Table 1). One striking observation was that all 116AG inoculated mice, whether they belonged to the short or long survival period population, inherited the long disease progression feature noticed upon first passage, compared to Wisc-1 inoculated mice [26]. For Wisc-1, in contrast, there was no significant difference in incubation period between first and second passage in these mice [26]. Surprisingly, Wisc-1 infection resulted in a significant decline in survival time on third passage in tg1536 mice (Figs 1 and S2A and Table 1). It also revealed the adaptation of 116AG-short to tg1536 mice since the survival period did not significantly change between second and third passage. Meanwhile, like Wisc-1, survival periods of 116AG-long inoculated tg1536 mice decreased. In addition, there were significant differences between survival times of mice inoculated with Wisc-1 and 116AG prions, both short and long, upon third passage. Wisc-1 inoculated mice still had a significantly shorter survival time compared to mice inoculated with either of the 116AG prions (Figs 1 and S2A). Also, mice inoculated with 116AG-short displayed significantly shorter survival periods than those inoculated with 116AG-long (Figs 1 and S2A and Table 1). As observed in the first and second passages [26], mice inoculated with 116AG prions upon third and fourth passages, both short and long, all exhibited a longer disease progression compared to mice inoculated with Wisc-1.

**Table 1:** Transmission of WTD isolates in different rodent models.

Animal model	Passage	CWD isolates (mean survival time in days ± SD)				Comments
		Wisc-1	116AG		H95^+^	
			short	long		
tg1536	1^st^	241 ± 20 (9/9) ^a^	301 ± 26 (10/10) ^a^		-	^a^ Data published in (Hannaoui, Amidian et al. 2017)
	2^nd^	248 ± 26 (5/5) ^a^	213 ± 38 (5/5) ^a^		-	^a^ Data published in (Hannaoui, Amidian et al. 2017)
	3^rd^	170 ± 23 (5/5)	199 ± 7 (5/5)	229 ± 11 (5/5)	-	116AG-short and -long have been inoculated separately
	4^th^	188 ± 28 (5/5)	226 ± 19 (5/5)	227 ± 30 (5/5)	-	
tg60	1^st^	> 600 (0/10) ^b^	634 ± 40 (14/14)		485 ± 5 (5/5)	^b^ Data published in (Velasquez, Kim et al. 2015)
Bank Vole	1^st^	300 ± 52 (3/5) *	> 700 (0/6)		-	* Two voles had died from intercurrent disease at 100 dpi and have been tested negative in RT-QuIC and western blot
	2^nd^	146 ± 15 (6/6)	-		-	
Syrian Golden hamsters	1^st^	652.6 ±.5 (3/8) ^c and^ **	638 (1/12) ***		-	^c^ Data published in (Herbst, Velasquez et al. 2017) ** 3/8 animals displayed clinical signs prior to experimental endpoint ** 8/8 animals were PrP^res^ positive ➔All animals had migration pattern at 21 kDa (classic) *** 1/12 animals displayed clinical signs prior to experimental endpoint *** 10/12 animals were PrP^res^ positive ➔ 7/10 had migration pattern at 21 kDa (classic) ➔ 3/10 had migration pattern at 20 kDa (low)

To further characterize adaptation of these isolates to tg1536 mice, we performed a fourth passage, upon which all WTD prions had adapted to their host with all isolates having similar survival periods between third and fourth passage. There was, however, still a significant difference between Wisc-1 inoculated mice and 116AG-short inoculated mice, with Wisc-1 inoculated mice having the shortest survival period. 116AG-short and -long inoculated mice did not have a significant difference in incubation periods upon fourth passage. Both 116AG-prions maintained the long disease progression upon fourth passage. Terminally sick tg1536 mice all showed comparable accumulation of PrP^res^ in the brain, regardless of the inoculated prions (S2B Fig).

We also assessed the PK resistance of the different prions, Wisc-1, 116AG-short and -long, throughout the different passages to verify whether the features observed in the original brain homogenates that were maintained in the first passage were still upheld [26]. In fact, PK resistance of the two WTD isolates showed that 116AG prions were less resistant to PK when compared to Wisc-1 prions, and that was also retained upon first passage [26]. Upon serial passages in tg1536 mice (2^nd^– 4^th^ passage), there was a significant difference between Wisc-1 and 116AG (Fig 1C). Wisc-1 prions were different from 116AG-short and 116AG-long prions. Remarkably, 116AG-short prions were also different from 116AG-long upon the subsequent passages (Fig 1C). 116AG-long prions were constantly less PK resistant than 116AG-short, indicating that the dominant conformer in the original isolate was probably the 116AG-long prions.

Next, we aimed to verify that the differences observed in tg1536 mice, in particular with regards to longer survival times of mice inoculated with the 116AG isolate, were due to the strain properties of each of the prions and not the amount of PrP^Sc^ in the original WTD isolates. While we observed no remarkable differences between the PrP^res^ amounts present in the brain homogenates of Wisc-1 and 116AG original isolates [26], the question of total PrP^Sc^ amount was still pending. A sandwich ELISA assay allowed for the quantification of total PrP^Sc^ in both WTD isolates. The ELISA assay demonstrated that total PrP^Sc^ in the WTD-116AG isolate is 2-fold higher than in the Wisc-1 isolate (S3 Fig). Therefore, we exclude the possibility that the PrP^Sc^ abundance, in this model, played a role in increased incubation period, disease transmission and/or clinical progression.

In summary, serial transmissions of WTD isolates in tg1536 mice strongly indicate that the 116AG isolate harbors, at least, one strain that is distinct from Wisc-1.

### Transmission in tg60 mice confirms that the 116AG isolate contains a distinct and new CWD strain

**Fig 2:**
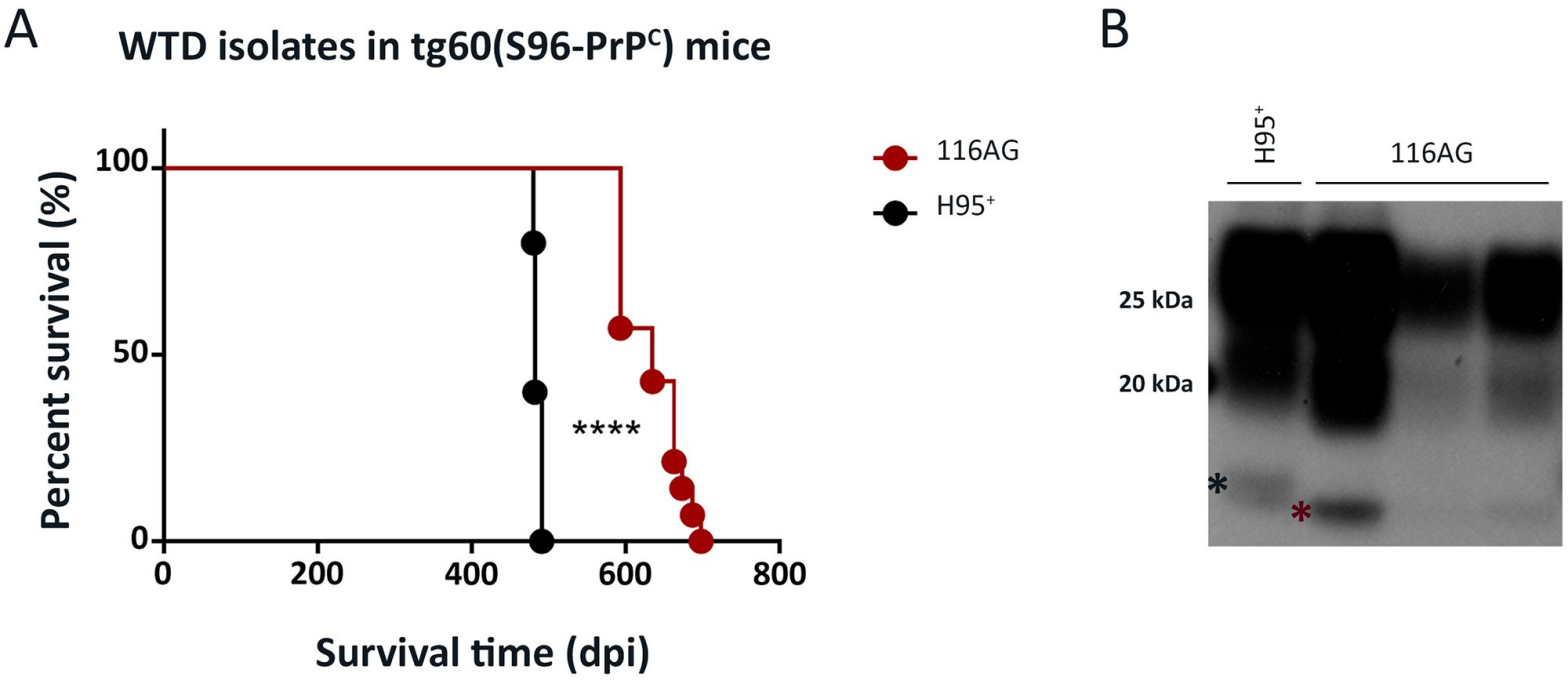
Transmission of the 116AG isolate to transgenic tg60 mice expressing S96-deer PrP. Tg60 mice, expressing S96-deer PrP at 0.7fold, were inoculated with 20 ul of 1% (w/v) brain homogenate containing 116AG or H95^+^ prions. (A) A Kaplan-Meier curve depicting the survival times of tg60 mice inoculated with 116AG or H95^+^ strains. ****p <0.0001, statistical differences were evaluated using a log-rank (Mantel-Cox) test. (B) Western blot of brain homogenates from mice inoculated with, H95^+^ (lane 1) or 116AG (lanes 2–4) prions after PK digestion. PrP^res^ was detected using the anti-prion monoclonal antibody BAR224. The asterisks show the migration profile, slow (black asterisk) and rapid (red asterisk), of the non-glycosylated bands.

We compared the transmission properties of Wisc-1 and 116AG prions in tg60 mice, a transgenic line initially thought to be resistant to CWD [36,37]. The tg60 mouse line expresses S96-deer PrP^C^ at a level 30% lower than the physiological level. This mouse line has been shown to be susceptible to the H95^+^ CWD strain [25,38]. Although Wisc-1 prions are readily transmitted into wt-deer PrP^C^ expressing mouse lines, tg1536 ([26]; Figs 1 and S2) and tg33 [25,38], it did not result in a productive infection in tg60 mice [25,38]. However, when the 116AG isolate was inoculated into tg60 mice, these mice developed clinical disease and succumbed with an average survival period of 633 dpi (Fig 2A). The survival time of 116AG inoculated tg60 mice was significantly longer than that of H95^+^ inoculated tg60 mice (Fig 2A). Western blot analysis of PK-treated PrP^Sc^ from brain homogenates of tg60 mice inoculated with 116AG or H95^+^ prions revealed that they had different biochemical signatures, with H95^+^ tg60 resistant-PrP^Sc^ having a slower migrating unglycosylated band than the 116AG tg60-PrP^Sc^. This was observed using anti-PrP antibodies with epitopes recognizing either the N-terminal (9A2) or the core of PrP^res^ (sha31 and BAR224; Figs 2B and S4).

### 116AG prions failed to replicate and induce disease in bank voles

**Fig 3:**
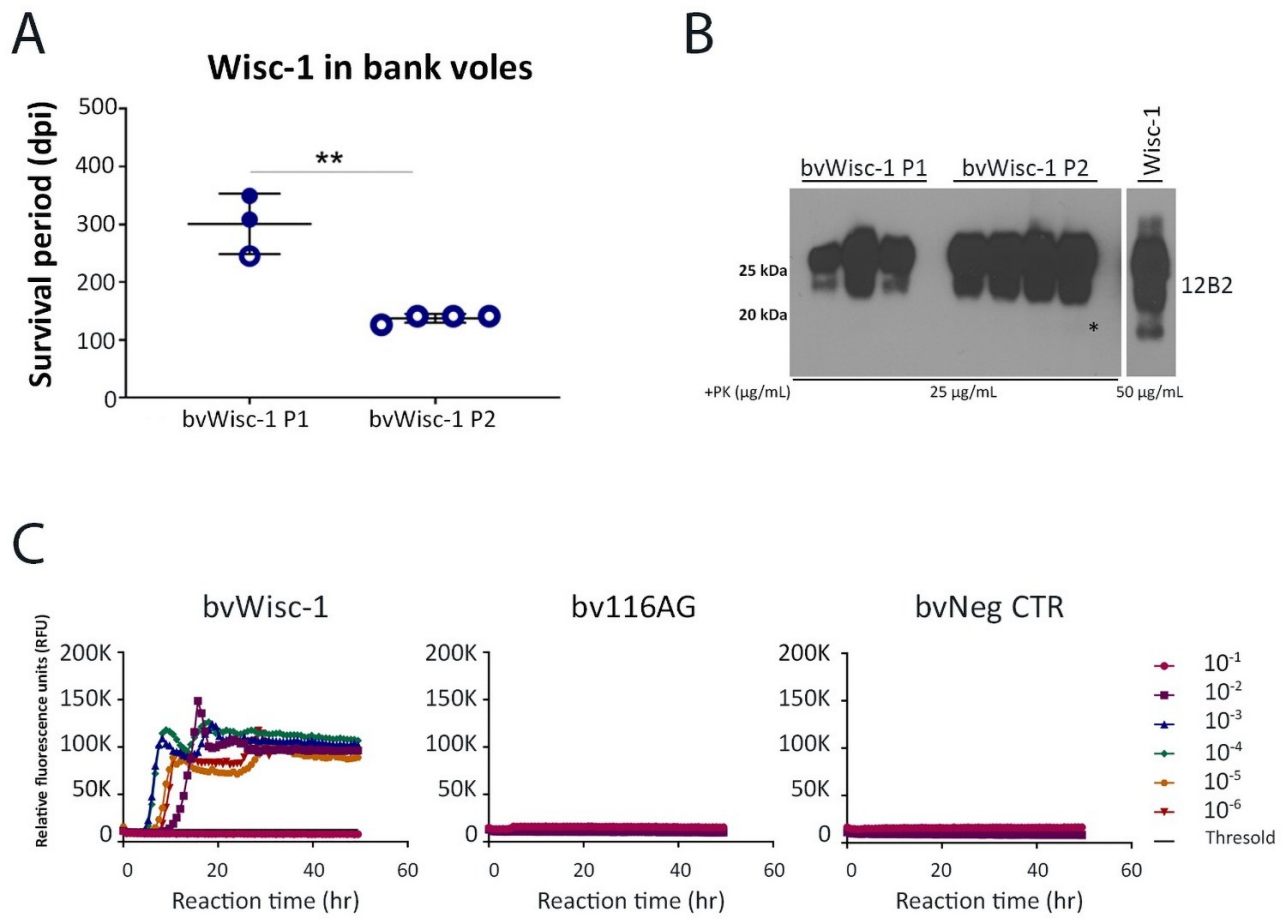
Transmission of Wisc-1 or 116AG prion isolates to bank voles. Inoculations of bank voles were performed through the intracerebral route using 20 ul of 1% (w/v) brain homogenate. (A) Survival times of bank voles upon inoculation with Wisc-1 prions upon first and second passage. None of the animals inoculated with 116AG prions showed prion disease signs upon experimental endpoint (700 dpi). **p <0.01, statistical analysis was performed using an unpaired student’s t-test. (B) Western blot using mAb 12B2 (aa 93 to 97) depicting PrP^res^ in brain homogenates of bank voles inoculated with Wisc-1 upon first passage (lanes 1–3), second passage (lanes 5–8) and Wisc-1 original isolate (lane 10; the illustrated blot is less exposed than that of 1^st^ and 2^nd^ passage PrP^res^). (C) RT-QuIC reactions were seeded with serially diluted (10^−1^ to 10^−6^) brain homogenates from bank voles inoculated with Wisc-1 (left panel) and 116AG (center panel) prions using rPrP bank vole substrate. Negative control is shown on the right panel. Fluorescence was measured every 15 min. The y-axis represents the relative fluorescence units (RFUs) and the x-axis the reaction time (hours). Each curve represents a different dilution and mean values of four replicates were plotted for each dilution. Reactions were positive when the mean RFU crossed the threshold (determined by averaging the RFUs of the negative control +5 SD).

We intracerebrally challenged bank voles, known to be susceptible to most prion strains, including CWD isolates [39–43], with Wisc-1 and 116AG prions. Wisc-1 inoculated bank voles had a 100% attack rate with an average survival time of 300 dpi upon first passage (Fig 3A). Western blot analysis of PrP^res^ from brain homogenates of Wisc-1 inoculated bank voles (bvWisc-1) showed PK-resistant PrP^Sc^ with a mostly undetectable non-glycosylated band compared to the Wisc-1 deer isolate when mAb 12B2 recognizing aa 93–97 or SAF84 binding to aa 167–173 were used (Figs 3B and S5). With mAbs 9A2 (aa 102–104) or Sha31 (aa 143–153) we were able to detect faint signals of the non-glycosylated band (S5 Fig). Upon second passage, bank voles inoculated with Wisc-1 displayed a significant decrease in their survival times with an average of 137 dpi (Fig 3A) indicating adaptation of Wisc-1 to its new host. The glycopattern of PrP^res^ upon second passage, was characterized by a very faint non-glycosylated band, thus resembling the first passage PrP^res^ in bank voles more than the Wisc-1 original isolate. Interestingly, bank voles inoculated with 116AG prions did not exhibit clinical signs up to 700 dpi (experimental endpoint). Brain homogenates of 116AG inoculated bank voles were tested by RT-QuIC, which did not reveal any seeding activity, while Wisc-1 brain homogenates resulted in positive seeding activity up to a 10^−5^ BH dilution in the RT-QuIC assay (Fig 3C).

All together, these results strongly support that the 116AG isolate contains CWD strains distinct from Wisc-1. Strikingly, it is also different from other characterized strains, such as H95^+^.

### 116AG prions constitute a mixture of new and distinct substrains

Based on the results obtained by serial transmission of the 116AG isolate into tg1536 mice ([26]; Fig 1), we suspected the pre-existence of two substrains within the 116AG original isolate.

**Fig 4:**
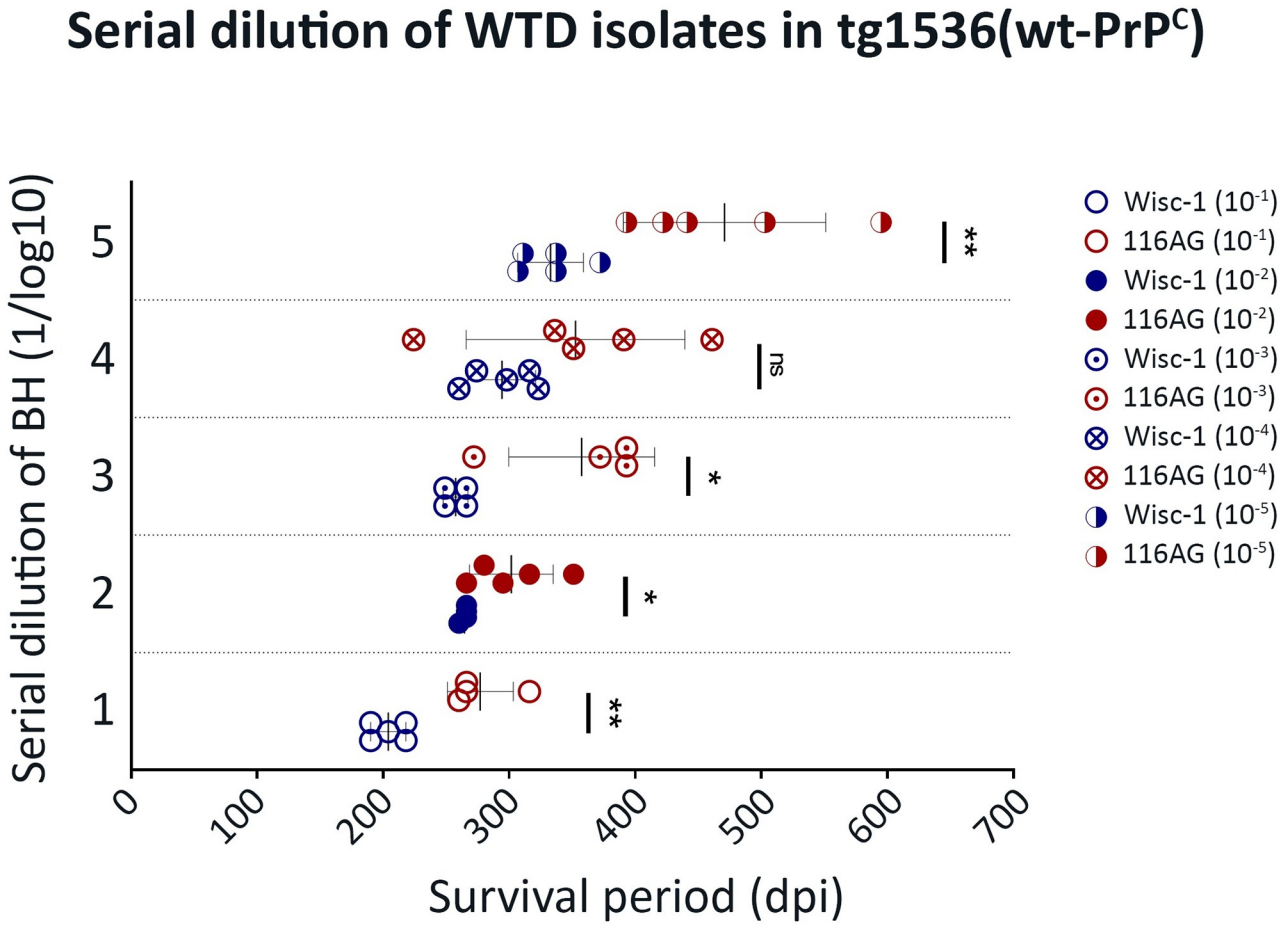
Inoculation of tg1536 mice with serial dilutions of Wisc-1 and 116AG. The graphs recapitulate the survival times of tg1536^+/+^ mice intracerebrally inoculated with 20 ul of serial tenfold dilutions of Wisc-1 (blue) and 116AG (red) isolates. An unpaired student’s t-test was used to compare between the different groups (*p <0.05, **p <0.01, ns p >0.05).

Biological cloning by inoculation of serially diluted brain homogenates has been used extensively in the past to corroborate whether the emergence of a new strain is the result of the serial passage of a known strain in a new host, or the pre-existence of this strain within a mixture of substrains at a minor level [32,44,45]. We intracerebrally inoculated 10-fold serially diluted brain homogenates of the 116AG isolate and Wisc-1 isolate into tg1536 mice (Table 2). In all groups of mice, the attack rate was 100% and biological cloning was not achieved; however, the results of this bioassay were very informative. The survival time differences between Wisc-1 and 116AG inoculated mice were still observed and significantly different for all inocula dilutions, except for the 10^−4^ dilution (Fig 4). Wisc-1 inoculated mice showed an expected increase in the survival time of mice from 10^−1^ to 10^−5^ dilutions, except for dilutions 10^−2^ and 10^−3^ where no statistical difference in the survival was observed (Fig 4 and Table 2). Furthermore, survival periods of 116AG inoculated mice strongly supported the presence of a mixture of strains in the 116AG isolate. The survival time differences between short and long incubation individuals became more prominent with increasing dilutions (Fig 4). For instance, mice inoculated with the 10^−4^ BH dilution of the 116AG isolate resulted in the shortest survival time observed across all different dilutions, thus, resulting in an average of survival in this dilution that was even shorter than 10^−3^ dilution, but the difference was not statistically significant. Yet, the relatively slower disease progression in mice inoculated with the 116AG isolate was still striking for both groups of mice (short and long) compared to the rapid disease progression upon inoculation with Wisc-1.

**Table 2:** Inoculation of tg1536 mice with serially diluted WTD isolates.

Animal model	Passage	Dilution	CWD isolates (mean survival time in days ± SD)	
			Wisc-1	116AG
tg1536	1^st^	10^−1^	204 ± 14 (5/5)	277 ± 26 (5/5)
	1^st^	10^−2^	264 ± 3 (5/5)	301 ± 33 (5/5)
	1^st^	10^−3^	257 ± 10 (5/5)	357 ± 58 (5/5)
	1^st^	10^−4^	294 ± 27 (5/5)	352 ± 86 (5/5)
	1^st^	10^−5^	332 ± 26 (5/5)	440 ± 46 (5/5)

**Fig 5:**
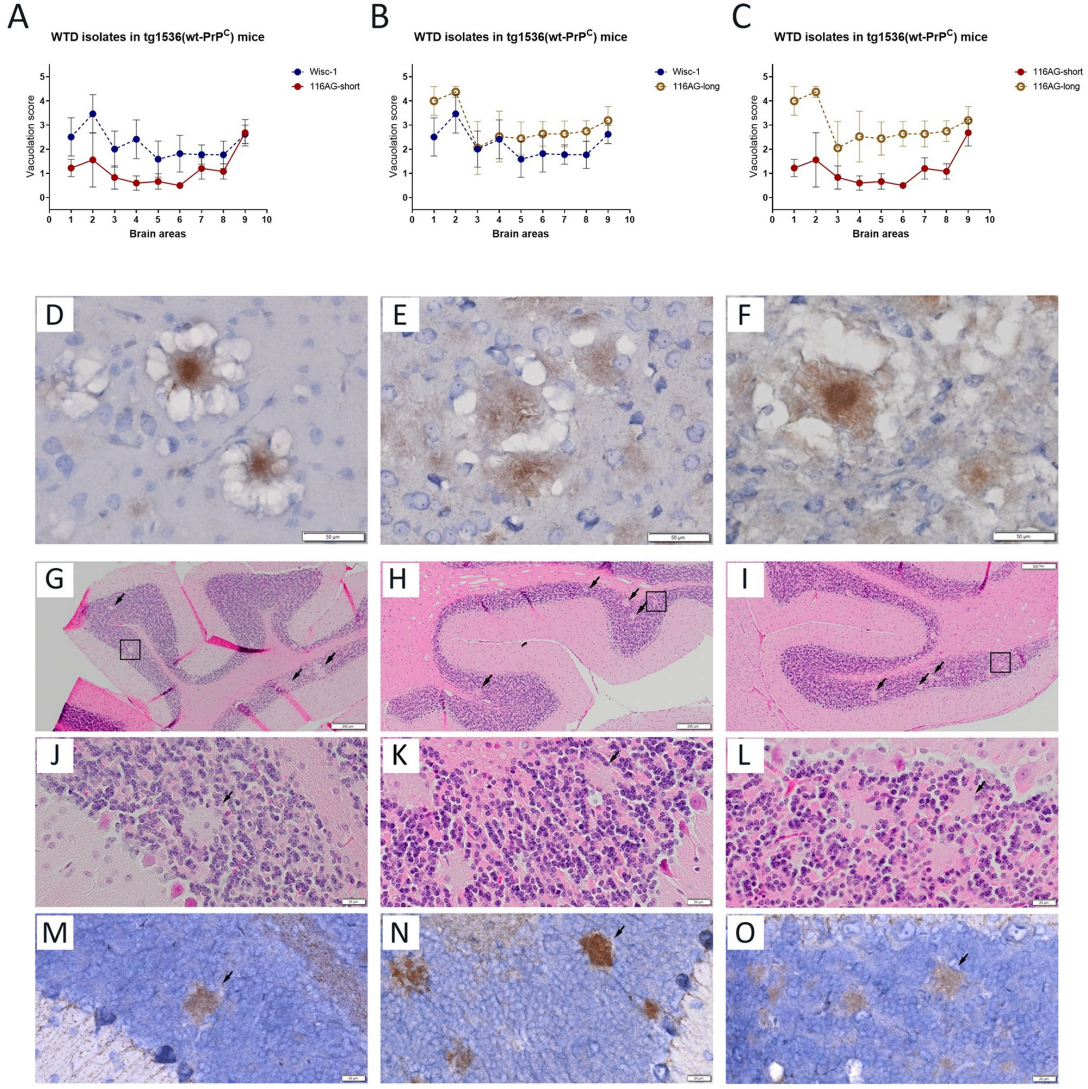
Strain characterization of tg1536^+/+^ mice inoculated with Wisc-1 and 116AG isolates. Neuropathological profiles of brains of tg1536^+/+^ animals inoculated with 10^−4^ dilution of Wisc-1 and 116AG prions, respectively, were determined. Brain vacuolation profiles comparing (A) 116AG-short and Wisc-1 inoculated mice, (B) 116AG-long and Wisc-1 inoculated mice, and (C) 116AG-short and -long inoculated mice. The y-axis represents vacuolation scores on a range of 0 to 5 in terms of spongiform changes (0 is none and 5 is severe). The x-axis represents the brain areas scored (1 frontal cortex, 2 basal ganglia, 3 parietal cortex, 4 hippocampus, 5 thalamus, 6 hypothalamus, 7 midbrain, 8 cerebellum, 9 medulla/pons). The scoring was performed in a blinded manner at least six times (range 6–8). Each curve represents each group, and each data point is the mean vacuolation score ± standard deviation. (D-F) Presence of florid plaques after PrP^Sc^ immunostaining using 12B2 mAb in the frontal cortex brain sections of mice inoculated with (D) Wisc-1, (E) 116AG-short and (F) 116AG-long prions. (G-O) Presence of unicentric plaques in the granular layer of the cerebellum of mice inoculated exclusively with 116AG-long. (G-I) H&E staining of three representative brains from mice inoculated with serially diluted brain homogenate of 116AG-long prions (10^−3^, 10^−4^ and 10^−5^). The unicentric plaques are indicated with black arrows and black squares. (J-L) H&E staining showing unicentric plaques highlighted by squares at a higher magnification. (M-O) PrP^Sc^ immunostaining using 12B2 mAb of the unicentric plaques in the granular layer of the cerebellum at a higher magnification. Scale bars indicate 20 μm and 50 μm for high magnification and 200 μm.

We have seen the dramatic difference in survival periods in the 116AG inoculated groups as an advantage. Thus, we explored neuropathological differences, using from each inocula dilution the mouse with the shortest incubation period, e.g. 116AG-short (n = 5), and the mouse with the longest incubation periods, e.g. 116AG-long (n = 5), and compared them to mice inoculated with Wisc-1 (Fig 5A–5C). Vacuolation scoring upon H&E staining ascertained the presence of different lesion profiles when 116AG-short and -long mouse brains were compared to each other (Fig 5C). In the brains of the 116AG-short mice, basal ganglia, hippocampus, thalamus, and hypothalamus regions were poorly affected. Frontal, parietal and cerebral cortex formations and mid brain displayed fairly mild vacuolization while the medulla/pons region showed a more severe spongiosis (Fig 5). In contrast, the lesion profile of 116AG-long mice was characterized by a significantly more severe vacuolization in most areas, with evident peaks in the basal ganglia and frontal cortex formations. All studied areas were statistically different from that of the 116AG-short group of mice.

To determine whether either of the 116AG substrains is similar to Wisc-1, we compared the lesion profiles of 116AG short and long, respectively, to that of Wisc-1 inoculated mice (Fig 5A and 5B). Lesion profiles of Wisc-1 inoculated mice are clearly distinct from those depicted for either of the 116AG prions (short and long). The Wisc-1 lesion profile was statistically different from 116AG-short in all studied area with the exception of the medulla (Fig 5A). It was also statistically different from 116AG-long in most of studied areas, with the exception of the parietal cortex and hippocampus (Fig 5B). In the frontal cortex and basal ganglia formations of most of the mice, we observed the presence of PrP plaques surrounded by areas of spongiform change, namely florid plaques (Fig 5D–5F), regardless of the inoculum or the dilution of the brain homogenate. Interestingly, we noticed the presence of unicentric plaques (Kuru-like plaques) in the granular layer of the cerebellum exclusively in mice inoculated with 116AG prions displaying the longest survival times (Fig 5G–5O) but not in those with short or intermediate survival time, nor in mice inoculated with the Wisc-1 isolate (S6 Fig). These plaques were not related to PrP^Sc^ deposits arising due to longer incubation periods, but rather to features of the 116AG-long prions. In fact, even mice with a comparably long incubation period from Wisc-1 and 116AG-short in the most diluted groups (10^−5^) did not show this kind of deposits.

**Fig 6:**
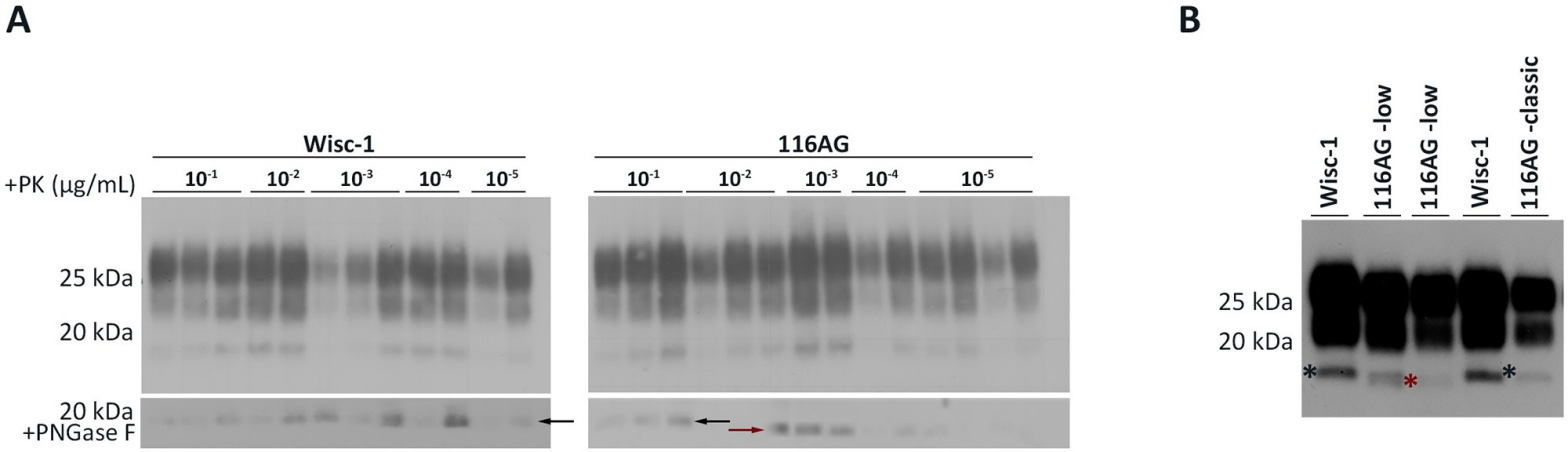
Different migration patterns of unglycosylated PrP^res^ are associated with 116AG prions. (A) Western blot of tg1536^+/+^ mice intracerebrally inoculated with 10fold serial dilutions of Wisc-1 reveals indistinguishable banding pattern either with (A; lower left panel) or without (A; upper left panel) PNGase F treatment after PK digestion, using 4H11 mAb. 116AG inoculated tg1536^+/+^ mice reveal a difference in the electrophoretic mobility (classic or low) in some samples after treatment with PNGase F and PK digestion (A; lower right panel). (B) The western blot depicts the different migration patterns of Syrian golden hamsters inoculated with Wisc-1 or 116AG prions, using 3F4 anti-PrP mAb. While Wisc-1 inoculated SGH always harbours the classic profile, 116AG inoculated animals show either the classic or the low migration. The asterisks/arrows show the migration profile, slow (black) and rapid (red), of the non-glycosylated bands.

Western blot analyses of PK-resistant PrP^Sc^ from brain homogenates of mice inoculated with 10-fold serially diluted Wisc-1 showed a typical three banding pattern indistinguishable from that of mice inoculated with 10-fold serially diluted 116AG prions after PK digestion (Fig 6A, upper panels). We next used PNGase F digestion to remove the N-linked glycans on PK-resistant PrP^Sc^ and analyzed the samples by western blot (Fig 6A, lower left panel). The results showed a homogeneous banding pattern in mice inoculated with the Wisc-1 isolate, referred to as classic. In samples from 116AG inoculated mice, PK-resistant fragments with different electrophoretic mobility were observed (Fig 6A, lower right panel). A group of mice showed a slow migration profile like Wisc-1 (21 kDa, classic), while others displayed a slightly more rapid migration profile (20 kDa, Fig 6A, lower right panel).

### Transmission of 116AG to Syrian golden hamster model results in distinct biochemical signatures of 116AG PK-resistant PrP^Sc^

Wisc-1 and 116AG WTD isolates were intracerebrally inoculated into Syrian golden hamsters, and the clinical signs and PrP^Sc^ patterns were determined (Table 1). For Wisc-1 inoculated hamsters, out of 8 inoculated animals, clinical disease was observed in 3 animals with an average incubation period of 652 dpi ([46]; Table 1). PK-resistant PrP^Sc^ was observed in 5 of 8 inoculated hamsters, with a classic migration pattern for all 5 animals ([46]; Table 1 and Fig 6B). For 116AG prions, only 1 out of 12 inoculated hamsters, with an incubation period of 638 dpi, developed clinical disease. However, western blot analysis of brain homogenates revealed the presence of PK-resistant PrP^Sc^ in 10 out of the 12 inoculated hamsters (Table 1). Interestingly, 7 hamsters out of the 10 with a PrP^res^ signal showed a classic migration pattern, similar to that of Wisc-1 inoculated hamsters (classic; Fig 6B), while the other 3 hamsters positive for PrP^res^ exhibited a rapid migration pattern (Fig 6B and Table 1), comparable to the rapid migration pattern observed in tg1536 mice inoculated with 116AG prions after PNGase F treatment (20 kDa, Fig 6A).

Our results, all together, strongly suggest the existence of two different strains in the 116AG isolate that are distinct from each other, and from Wisc-1 and H95^+^ CWD strains.

## Discussion

In this study, we provide strong experimental evidence, from both homotypic and heterotypic transmission of natural CWD prions to transgenic mouse, bank vole and hamster models, that distinct strains can co-exist in brains of infected deer expressing allelic variants of PrP^C^ heterozygously. They can be propagated in a homotypic line or when the recipient carries a different *Prnp* gene sequence, from the same or different species (S1 Fig).

Although a number of studies have now described CWD strains [21,24,25,38,47,48], the diversity and emergence of these strains are still poorly documented. Two prevalent CWD prion strains were first described, referred to as CWD1 and CWD2, which displayed different clinical and neuropathologic profiles despite indistinguishable biochemical properties of PrP^Sc^ [24]. One major finding of this study was that elk isolates only contain one strain while deer isolates can harbor a mixture of the CWD1 and CWD2 strains [24]. This finding confirmed a previous study that raised the possibility of strain mixtures within CWD cervid brains [16], which was eventually attributed to the difference between elk (226E) and deer (226Q) PrP and strain mutation [24].

The *Prnp* coding sequence is very well conserved between cervid species, yet there are some species-specific key polymorphisms that are associated with lower attack rates and/or slower disease progression following CWD infection. Polymorphisms in cervid *Prnp* have been identified at codon 132 of the elk *Prnp* gene, encoding either methionine (M) or leucine (L) [49], equivalent to that of position 129 (M/Valine) in the human *PRNP* gene [50], which has significant effects on human prion disease presentation and susceptibility [51,52]. In WTD, polymorphisms at position 95 (encoding glutamine (Q) or histidine (H)), 96 (encoding glycine (G) or serine (S)) and 116 (encoding alanine (A) or G) have been identified [27]. Polymorphisms at position 138 (asparagine (N) or S) in caribou [53–57] and 225 (S or phenylalanine (F)) in mule deer have been also reported [58–60]. These well-characterized polymorphisms appear to play an important role in favoring the emergence of new and stable CWD strains [17,25,26,38,46,47,61,62].

Here, we demonstrate that 116AG prions are distinct from Wisc-1, based on differences in survival times (Fig 1) and disease progression in tg1536 mice, as well as different biochemical properties ([26]; Figs 1C and 6B) and neuropathological features (Fig 5A–5C). When the WTD isolates were inoculated in bank voles (Fig 3A and 3B), Wisc-1 inoculated voles developed prion disease, which adapted to voles upon second passage, while 116AG inoculated bank voles did not develop prion disease, nor accumulate PrP^res^ *in vivo* or show a positive seeding activity in RT-QuIC reactions seeded with brain homogenates (Fig 3C). The reverse occurred upon infection of tg60 mice with 116AG (Fig 2A) resulting in disease, but not Wisc-1 [25,38]. Because only H95^+^ has previously induced clinical disease in tg60 mice, the different migration patterns of 116AG and H95^+^ PrP^res^ in tg60 mice provided strong evidence that these strains are different from one another.

Hence, we postulate that 116AG prions are different from Wisc-1/CWD1, CWD2 (asymmetrical distribution of PrP^Sc^ in the brain) and H95^+^ strains, and, in deer, existed as a strain mixture. The 116AG-long strain was observed in PrP-overexpressing tg1536 mice upon first passage, and the 116AG-short strain emerged in the second passage (Figs 1 and S2). Up to the 4^th^ passage, all inoculated mice (Wisc-1, 116AG-short and-long) adapted to tg1536 illustrated by a stabilized survival period (Figs 1 and S2), yet differences between Wisc-1 inoculated mice and 116AG inoculated mice were still perceptible. The difference in survival time between 116AG-short and -long was no longer significant. This may be due to the presence of residual short- or long-PrP^Sc^ in the 116AG-long or -short groups, respectively, leading to animals having short and long survival times in both groups, and may therefore result in a more scattered population, which we observe in the 4^th^ passage. An alternative explanation is that these strains are unstable in this model and converge into a state of a similar strain upon passaging. However, biochemical differences (resistance to PK) were still observed between Wisc-1, 116AG-short and 116AG-long (Fig 1C). In fact, these biochemical features were retained from original deer isolates through 4^th^ passage ([26], Fig 1C). What caught our attention was the fact that 116AG-long prions always displayed less PK resistance compared to Wisc-1 and 116AG-short, thus we conclude that the major conformer in the original 116AG isolate was 116AG-long.

The means by which strain diversity is encoded by PrP^Sc^ is not clear. It is also not clear if strains emerge from mixtures of substrains or if a unique strain evolves under certain conditions (i.e., heterologous transmission). There are two scenarios, one featuring emergence of a new strain not present in the original isolate upon passage into a distinct host, which was the case for drowsy (DY) and hyper (HY) hamster-adapted TME strains [35,45,63,64]. In a second scenario, different prion substrains co-exist within one isolate, which has been widely described in scrapie as well as in human prion diseases. Indeed, sCJD patients harbored both type 1 and type 2 PrP^res^ in the brain demonstrating that at least two sCJD prion strains can be present in a single isolate from one patient [65,66]. In our study, the second scenario seems more plausible. The inoculation of serially diluted 116AG isolate in tg1536 mice showed a more heterogenous population, with more widespread survival periods within the same dilution. Remarkably, the more diluted the inoculum was, the wider this difference was (Fig 4). One of the most striking differences was observed in mice inoculated with 10^−4^ brain homogenate dilution with one mouse having the shortest survival period, 224 dpi, of all 116AG inoculated mice, including those inoculated with lower dilutions. This mouse’s incubation period was shorter than mice inoculated with Wisc-1 prions within the same dilution (10^−4^), and other less and more concentrated dilutions (10^−2^, 10^−3^ and 10^−5^), and it was comparable to the incubation times of Wisc-1 inoculated mice in the most concentrated homogenate (10^−1^). Mice inoculated with the 10^−4^ brain homogenate dilution of the 116AG isolate also displayed the largest difference between the shortest (224 dpi) and longest (461 dpi) survival periods within this group of mice (Fig 4). Previously, transmission of CWD in tg1536 mouse model had shown an unstable strain propagation [24]. In fact, CWD1 or 2 propagated in deer-PrP^C^ context converged into a mixture of both strains, while it manifested either in CWD1 or CWD2 strains after transmission into elk-PrP^C^ overexpressing mice. This instability was attributed to the single primary structure difference between deer and elk PrP at codon 226. In contrast, our results suggested that transmission to the tg1536 mouse model [24] can in fact generate distinct strains like 116G-short and -long, e.g. in the 10^−4^ dilution and up to the 4^th^ passage.

Supporting evidence for pre-existing and distinct substrains in the 116AG isolate was obtained by comparing neuropathological profiles. The vacuolation scores of 116AG-short mice showed a different neuropathologic profile from that of 116AG-long mice (Fig 5C). More interestingly, neither of them had a neuropathologic profile resembling Wisc-1 (Fig 5A and 5B). In addition, we noted the presence of unicentric PrP plaques exclusively in the cerebellum of mice harboring 116AG-long prions (Fig 5G–5O).

Because we assume the presence of 116A-PrP^C^ derived PrP^Sc^ conformer in the 116AG prions, we expected the propagation of Wisc-1 prions in our models. However, the different neuropathological profiles proves that none of the 116AG substrains was similar to Wisc-1. Additionally, since voles inoculated with Wisc-1 all developed prion disease, the fact that none of the voles inoculated with 116AG prions developed prion disease or had detectable levels of PrP^Sc^ supports that Wisc-1 prions are absent from the 116AG deer isolate. Because the 116AG prions used in this study were from a field isolate, we ignore what strain was the source of its CWD infection. However, the vast majority of WTD are homozygous for the wild-type allele. Thus, we hypothesize that the source of infection, most likely, contained Wisc-1 prions. If this postulate is true, the presence of one allele encoding 116G-PrP was sufficient to overcome/inhibit the replication of Wisc-1 prions, and the conversion of the wt allele (116A) gave rise to a conformer that is different from Wisc-1.

Indeed, the nature of interaction/interference of the 116AG substrains with each other and with Wisc-1 has yet to be determined. Similar to previous studies [45,67], co-infection of Wisc-1, 116AG-long and -short at various brain homogenate ratios is required both *in vitro* and *in vivo* to determine the extent and nature of interference of these strains.

Our data add new insights into the existing variety of CWD strains. In fact, identifying and characterizing new CWD strains, in addition to revealing the influence of PrP primary structure on their properties, is critical for predicting the potential for transmission between species. The occurrence of vCJD in humans, a consequence of BSE transmission, provides evidence that the human species barrier to animal prion diseases is not impenetrable [14,68]. In particular, CWD, with its wide tissue distribution of infectivity, persistence in the environment, and geographical spread should be placed at the forefront of our concerns. Eventually, characteristics associated to CWD susceptibility of cervids expressing polymorphic PrP can lead to infected animals with longer incubation periods, potentially resulting in prolonged shedding of infectious prions into the environment as well as emergence of novel CWD substrains with unknown potential to infect other species.

## Material and methods

### Ethics statement

All animal use in this study strictly followed the guidelines of the Canadian Council for Animal Care. All experiments detailed in the study were performed in compliance with the University of Calgary Animal Care Committee under protocol number AC18-0047 and the Animal Care and Use Committee: Health Sciences at the University of Alberta Animal Care under protocol number AUP914.

Prior to inoculation and euthanasia, isoflurane was used as anesthetic at a concentration of 5% (flow rate of 0.8 L/min) for induction, and then lowered to 0.5–1% for maintenance of general anesthesia.

### CWD isolates

CWD isolates were prepared as 10% (w/v) brain homogenates in phosphate-buffered saline pH 7.4 (PBS; Life Technologies, Gibco) using either a dounce homogenizer or the MP Biomedicals fast prep-24 homogenizer (Fisher). Aliquots were stored at −80°C for further use. Wisc-1 isolate was obtained upon experimental oral infection of WTD [17,25], and 116AG isolate was provided by the Canadian Wildlife Health Cooperative (CWHC), Saskatoon, SK, Canada [26]. It was from a 5 years old wild WTD male that was reported to exhibit clinical signs (wasting syndrome) and later found dead and tested positive for CWD. No other PrP polymorphisms were found in this animal, except for codon 116. We also used the previously characterized H95^+^ CWD strain [25,38] for prion infection.

### Animal study

WTD isolates were transmitted to transgenic mouse lines expressing deer wt PrP^C^ (tg(CerPrP132M)1536^+/+^, referred to as tg1536^+/+^) or the WTD S96-PrP^C^ (tg60), Syrian golden hamsters and bank voles. Tg1536^+/+^ mice overexpress PrP in the brain about six to eightfold [22], and the tg60 mouse line expresses 30% less PrP^C^ than physiological levels [36,37]. We also used bank voles expressing a methionine at position 109 of the *Prnp* gene [39,43] and Syrian golden hamsters (Envigo). Bioassays were performed as described previously [25,26,38]. Briefly, weanling pups were anaesthetized and, unless otherwise stated (10-fold dilution series), inoculated with 1% WTD original or passaged brain homogenates (Wisc-1, 116AG and H95^+^) in the right parietal lobe using a 25 gauge disposable hypodermic needle. Each BH inoculum in the serial passages consisted of a pool of at least 3 characterized animals (mice or voles), with the exception of 3^rd^ passage 116AG-long in tg1536 mice, which was done using a single animal from the 2^nd^ passage. Inoculated animals were initially monitored weekly. Upon onset of clinical signs, they were monitored daily. At the experimental endpoint, mice and voles were exhibiting a rigid tail, rough coat, lack of balance, ataxia, hunched posture, and weight loss. Clinical hamsters were ataxic with retrocollis and presented a progressive lethargy. At this point, animals were anaesthetized before being euthanized by CO_2_ overdose. Brains were collected and either fixed in formalin or frozen at -80°C. Based on animal models, experimental termination endpoints were pre-determined.

Statistical analyses were performed using GraphPad Prism (version 7) software. For all transmission experiments, an unpaired student’s *t*-test was used. All mice used in this study (statistical analysis and graphs) were enclosed in a protocol of inclusion criteria determined by the experimental endpoint described above. A protocol of exclusion criteria was determined based on a humane endpoint of mice, which succumbed to intercurrent disease during the experiments. None of the mice subject to the exclusion criteria were used in the analyses.

### Anti-PrP antibodies

Detection of prion protein by western blot was performed using primary monoclonal mouse antibodies 4H11 ([69]; 1/500), BAR224 (aa 141 to 151; diluted at 1:10,000; Cayman Chemical), 9A2 (aa 102–104; diluted at 1:5000; Wageningen UR), 12B2 (aa 93–97; diluted at 1:1000; Wageningen UR), SAF84 (aa 167–173; diluted at 1:1000; Bertin-Bioreagent), sha31 (aa 143–153; diluted at 1:10000; Bertin-Bioreagent), and 3F4 (aa 109–112; diluted at 1:10000; a kind gift from Dr. Richard Rubenstein).

### Western blot

For PrP analysis in brain extracts of different animal models, brain homogenates prepared in PBS were digested with different concentrations of proteinase K (PK; Roche) for 1 hour at 37°C. The enzymatic reaction was terminated by the addition of 1X pefabloc proteinase inhibitor (VWR), then samples were denatured at 96°C for 10 min in 3X SDS (sodium dodecyl sulphate) sample buffer. For deglycosylation of PK-digested and denatured samples, PNGase F enzyme (Roche) was added to the samples according to the manufacturer’s instructions. Samples were resolved on 12% NuPAGE bis-tris gels (Life Technologies), and then electrophoretically transferred to PVDF membranes (Millipore, Ca). PVDF membranes were probed using anti-PrP monoclonal antibodies followed by horseradish peroxidase-conjugated goat anti-mouse IgG antibody (Sigma) and developed using ECL-plus detection (Amersham). Images were acquired on X-ray film (Super Rx; Fujifilm).

### RT-QuIC assay

The bank vole recombinant PrP used as a substrate in RT-QuIC reactions was prepared as described [26,70]. RT-QuIC was performed as described [26,70]. Briefly, reactions were set up in assay buffer containing 20 mM sodium phosphate (pH 6.9), 300 mM NaCl, 1 mM EDTA, 10 μM Thioflavin T and 0.1 mg/ml bank vole recombinant PrP substrate. Ninety-eight μl aliquots were added to the wells of a 96 well optical bottom plate (Nalge Nunc International). Quadruplicate reactions were seeded with 2 μl of brain homogenate (10%) from CWD-negative bank voles (negative control), Wisc-1 inoculated bank voles, or 116AG inoculated bank voles that were 10fold serially diluted in RT-QuIC seed dilution buffer (0.05% SDS in PBS). The plate was sealed with Nunc Amplification Tape (Nalge Nunc International) and placed in a BMG Labtech FLUOstar Omega fluorescence plate reader that was pre-heated to 42°C for a total of 50 hours with cycles of 1 minute double orbital shaking (700 rpm) incubation and 1 minute resting throughout the incubation. ThT fluorescence signals of each well were read and documented every 15 minutes; values were plotted as the average of quadruplicate reactions by using GraphPad Prism software.

### Vacuolation scoring

Brain tissues from mice inoculated with either Wisc-1 or 116AG serially diluted brain homogenates were formalin fixed (BDH chemicals) and paraffin embedded for histopathological analysis. Sagittal brain sections (6 μm) were cut and stained using hematoxylin and eosin (H&E; Leica) to evaluate the sections for spongiform changes and immunostained for PrP^Sc^ deposition using mAb 12B2 as described in [26]. Spongiform degeneration was assessed at nine different regions of the brain (frontal cortex, basal ganglia, parietal cortex, hippocampus, thalamus, hypothalamus, midbrain, cerebellum, and medulla/pons). Sections were scored on a scale of 0 (absence) to 5 (severe) for the presence and severity of spongiform degeneration as described previously [14,71]. The scoring was performed at least six times (range 6–8) in a blinded manner. The scores are reported as the mean ± SD and the values were plotted by using GraphPad Prism (version 7) software.

### Sandwich ELISA

#### Enrichment of PrP^Sc^

Fifty microliter (μL) aliquots of CWD-infected brain homogenate (10%, w/v) were dispensed into standard 2 mL microfuge tubes with screw caps and rubber O-rings. The aliquots were mixed with 1.9 mL of RiPa cell lysis buffer (150 mM NaCl, 1% NP-40, 0.25% Sodium deoxycholate, 1 mM EDTA, 50 mM Tris, pH7.4) and incubated at room temperature for 30 min. After centrifugation for 20 min at 20,000 *x* g, the sample separates into an insoluble pellet fraction (IPF) and a clarified supernatant fraction (SF). The SF was completely removed and discarded from each tube taking care to avoid disturbing the pellet. The intact IPF was re-suspended in 50 μL of 8 M guanidine hydrochloride (GdnHCl). The GdnHCl dissolved IPF samples was enriched for PrP^Sc^ in approximately same the volume of 10% (w/v) brain homogenate from which they were derived. For the negative controls, GdnHCl dissolved IPF samples from brain homogenates of uninfected WTD were prepared following the same method as described above.

#### PrP standard calibration

To create reference samples with standardized concentrations of PrP, purified recombinant mouse PrP23-231 (recMoPrP) was 2fold serially diluted in 8 M GdnHCl ranging from 2.5 to 0.039 μg/mL. These 7 different concentrations of recMoPrP were used as PrP standard calibration samples and assayed by adding 5 μL each standard in 150 μL reaction buffer (1% BSA in PBS) for each ELISA test-well.

#### Sandwich ELISA

ELISA strip plates (Santa Cruz, USA) were coated with 0.5 μg/well of a novel anti-PrP monoclonal antibody (mAb) D15.15 (Tang *et al*., manuscript in preparation) in PBS by overnight incubation at 4°C. The plates were blocked with 200 uL/well of 3% BSA in PBS for 2 hrs at room temperature (RT) and subsequently washed with TBST (TBS with 0.1% of tween 20). Five microliter of each PrP^Sc^ enriched samples and PrP standard were mixed with 150 μL of reaction buffer and added into the test-well in triplicate. The plate was incubated for 90 min at RT. After washing with TBST, the captured PrP was detected by incubation with an HRP-conjugated anti-PrP mAb N5 (Tang *et al*., manuscript in preparation) for 1 hour at RT. The plates were again washed with TBST and 100 μL of TMB substrate (Surmodics, USA) was added. After incubation for 20 min at RT in the dark, the absorbance was measured at 650 nm and used for PrP quantification. The average standard calibration OD values obtained by ELISA were used to plot an 8-parameter linear curve fit to the standards and then calculate the PrP concentrations for the test samples.

## Supporting information

S1 Fig Prion protein sequence alignment showing homology and polymorphisms between species. Protein alignment was performed in Geneious v10.2.6 (https://www.geneious.com) using the ClustalW algorithm. Amino acid numbering is based on the consensus sequence. Amino acid variants were added manually to each sequence and are shown in white boxes. β1, β2: first, second beta-strand; α1, α2, α3: first, second and third alpha-helix (based on mouse PrP numbering). 1. *Odocoileus virginianus*, Wt-deer PrP sequence; 2. *Odocoileus virginianus*, G116-deer PrP sequence; 3. *Odocoileus virginianus*, S96-deer PrP sequence; 4. *Mesocricetus auratus*, Syrian golden hamster PrP sequence, and 5. *Myodes glareolus*, bank vole M109-PrP sequence.
(TIF)

S2 Fig Serial transmission of Wisc-1 and 116AG isolates in tg1536^+/+^ mice. (A) Survival times of Wisc-1 (blue) and 116AG (red) groups in the first (circle), second (square), third (triangle) and fourth (diamond) passage. In the Wisc-1 group, there is a significant decrease in incubation time from the second to the third passage. In the 116AG group, there is a significant decrease in incubation time from the first to second passage, and in the second passage two populations with different survival times, denoted 116AG-short (filled shape) and 116AG-long (unfilled shape), emerged. Statistical analyses were performed using a student’s t-test, **p <0.01, ***p <0.001, ****p <0.0001, and ns p >0.05. (B) Representative western blot reveals the PrP^res^ profile in the brain of inoculated animals upon 4 passages alongside Wisc-1 and 116AG deer isolates. PrP^res^ was detected using the anti-prion monoclonal antibody 4H11.
(TIF)

S3 Fig Total PrP^Sc^ levels of WTD isolates using ELISA. Levels of PrP^Sc^, as determined by sandwich ELISA, are about 2 times higher in 116AG samples than in Wisc-1 samples. (TIF)

S4 Fig Different migration patterns of H95^+^ and 116AG prions in tg60 mice. Western blot of brain homogenates from mice inoculated with, H95^+^ or 116AG prions after PK digestion. The same samples have been loaded on the same gel twice, then cut into two to be probed with different mAbs. PrP^res^ was detected using the N-terminal anti-prion monoclonal antibody 9A2 (upper panel) and a central-region anti-prion monoclonal antibody sha31 (lower panel). The asterisks show the migration profile, slow (black asterisk) and rapid (red asterisk), of the non-glycosylated bands.
(TIF)

S5 Fig Transmission of Wisc-1 prion isolate to bank voles. Inoculations of bank voles were performed through the intracerebral route using 20 ul of 1% (w/v) brain homogenate. Western blot analyses were performed using mAbs (A) 9A2 (aa 102 to 104), (B) SAF84 (aa 167 to 173), and (C) Sha31 (aa 143 to 153) depicting PrP^res^ in brain homogenates of bank voles inoculated with Wisc-1 upon first passage (lanes 1–3), second passage (lanes 5–8) and Wisc-1 original isolate (lane 10). Results with different antibodies were obtained from individual western blots of the same samples.
(TIF)

S6 Fig Absence of unicentric plaques in the cerebellum of Wisc-1 and 116AG-short inoculated tg1536^+/+^ mice. H&E staining (A-B and E-F) and immunostaining of PrP (C-D and G-H) of brain sections of tg1536^+/+^ mice inoculated with Wisc-1 (A-D) or 116AG-short (E-H) confirms the absence of unicentric plaques in the cerebellum of these animals. Scale bars indicate 50 μm (B, D, F and H) as high magnification and 500 μm (A, C, E and F).
(TIF)
