# Supplementary figures and images for: New and distinct chronic wasting disease strains associated with cervid polymorphism at codon 116 of the Prnp gene
Source: PLoS Pathog. 2021 Jul 26;17(7):e1009795. doi: 10.1371/journal.ppat.1009795 (PMC8341689; doi:10.1371/journal.ppat.1009795)

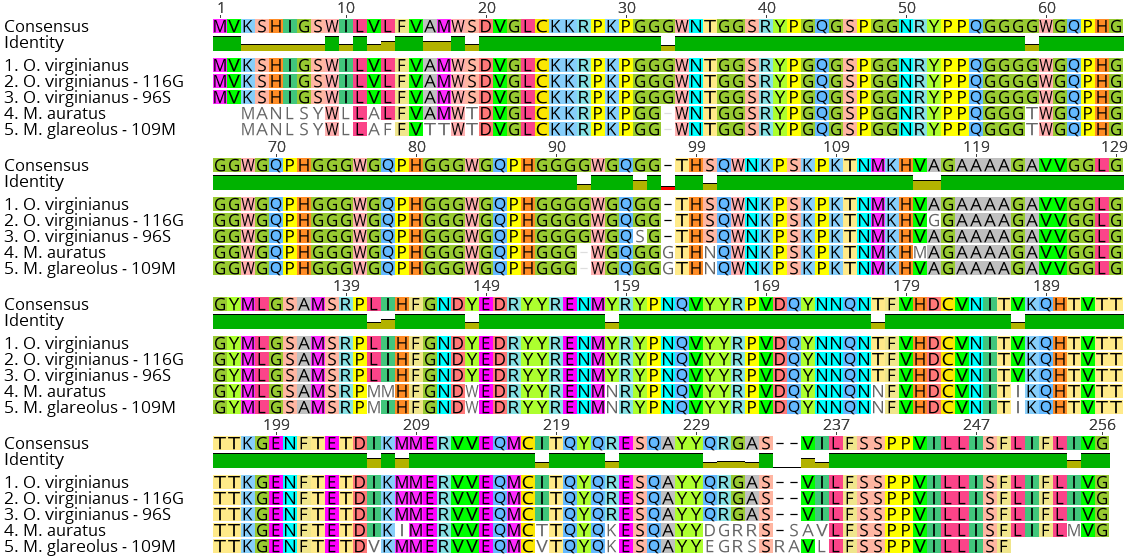

Supplement: S1 Fig — Protein alignment was performed in Geneious v10.2.6 (https://www.geneious.com) using the ClustalW algorithm. Amino acid numbering is based on the consensus sequence. Amino acid variants were added manually to each sequence and are shown in white boxes. β1, β2: first, second beta-strand; α1, α2, α3: first, second and third alpha-helix (based on mouse PrP numbering). 1. Odocoileus virginianus, Wt-deer PrP sequence; 2. Odocoileus virginianus, G116-deer PrP sequence; 3. Odocoileus virginianus, S96-deer PrP sequence; 4. Mesocricetus auratus, Syrian golden hamster PrP sequence, and 5. Myodes glareolus, bank vole M109-PrP sequence. (TIF) [file ppat.1009795.s001.tif]

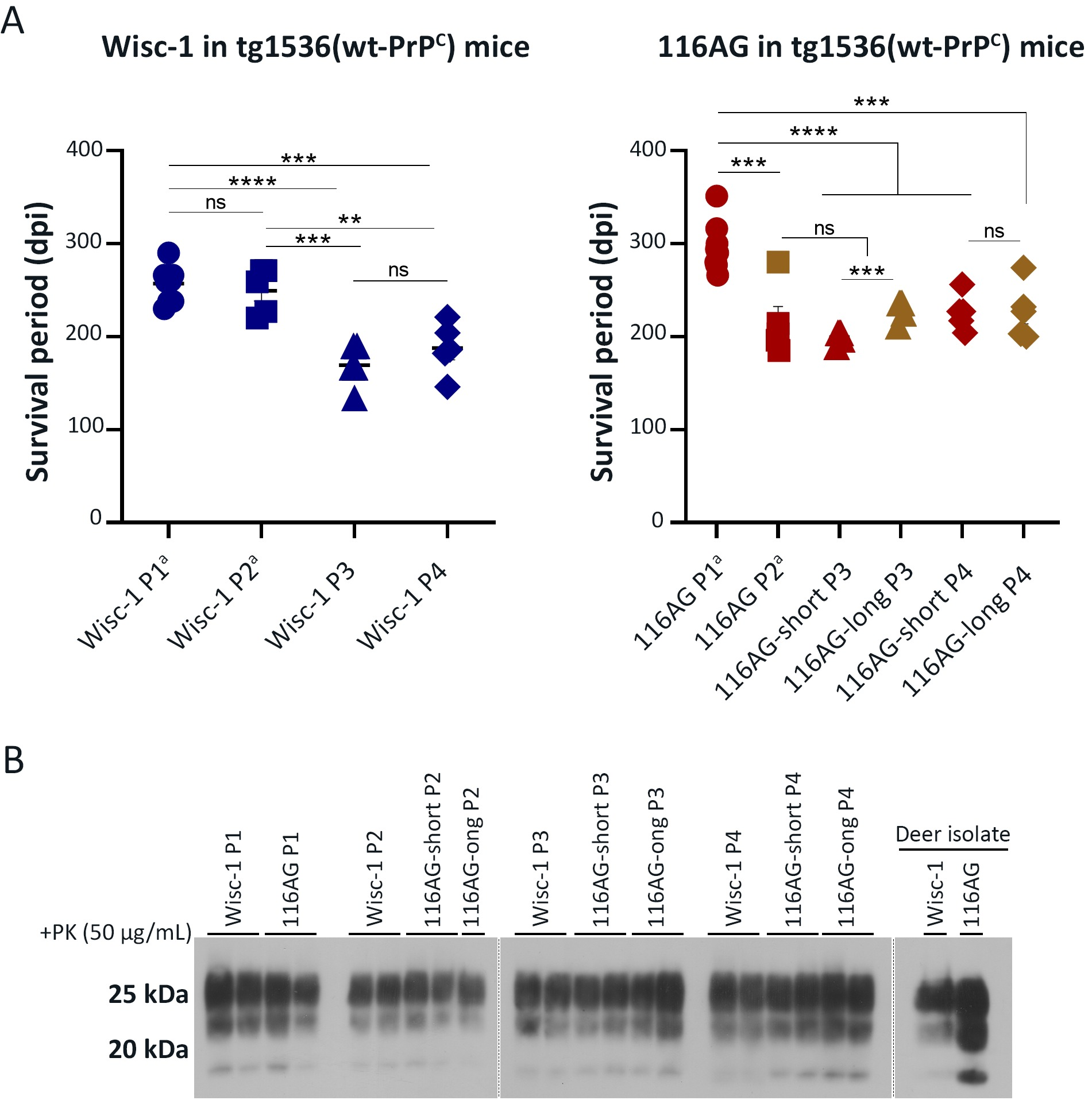

Supplement: S2 Fig — (A) Survival times of Wisc-1 (blue) and 116AG (red) groups in the first (circle), second (square), third (triangle) and fourth (diamond) passage. In the Wisc-1 group, there is a significant decrease in incubation time from the second to the third passage. In the 116AG group, there is a significant decrease in incubation time from the first to second passage, and in the second passage two populations with different survival times, denoted 116AG-short (filled shape) and 116AG-long (unfilled shape), emerged. Statistical analyses were performed using a student’s t-test, **p <0.01, ***p <0.001, ****p <0.0001, and ns p >0.05. (B) Representative western blot reveals the PrPres profile in the brain of inoculated animals upon 4 passages alongside Wisc-1 and 116AG deer isolates. PrPres was detected using the anti-prion monoclonal antibody 4H11. (TIF) [file ppat.1009795.s002.tif]

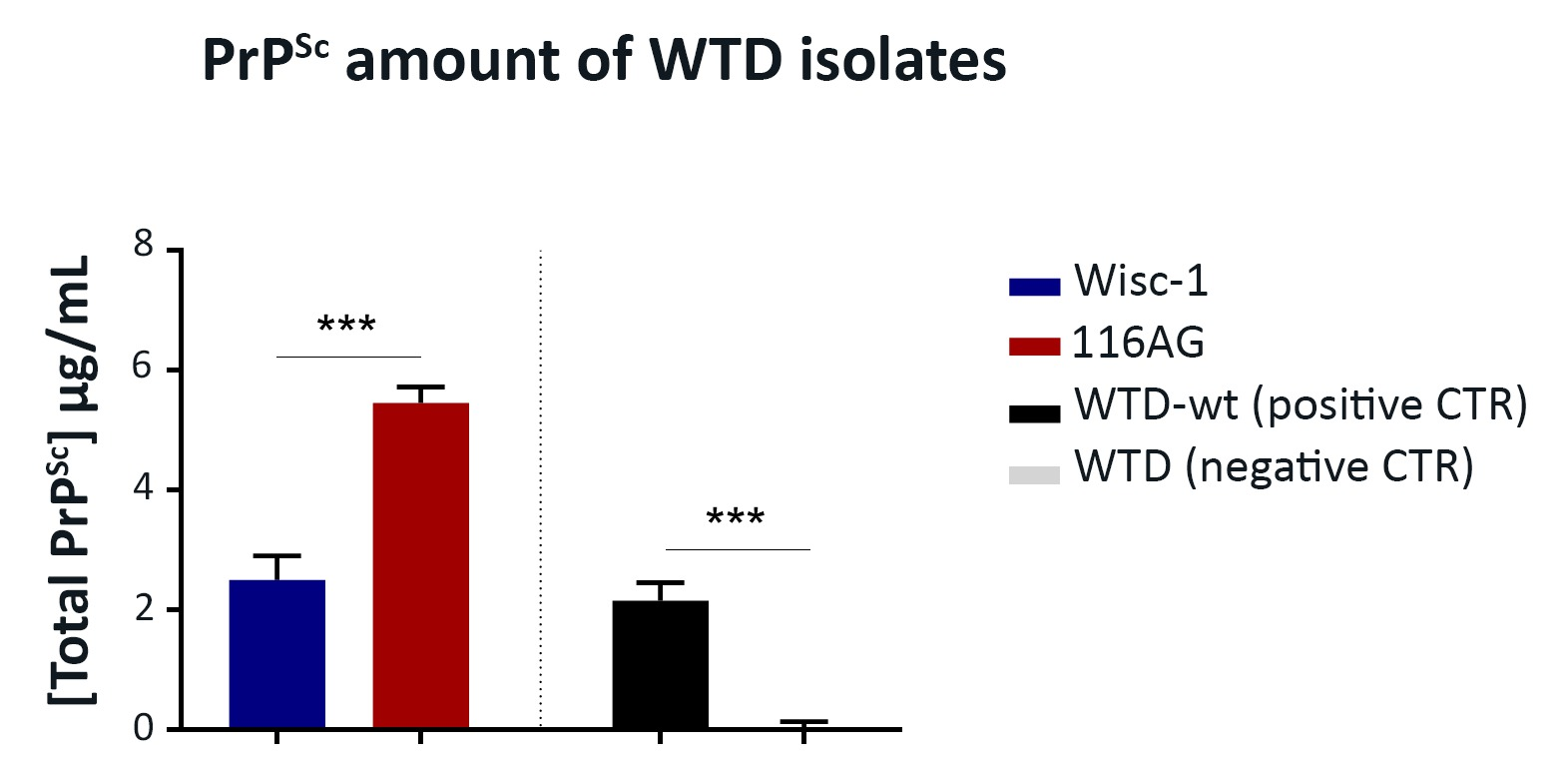

Supplement: S3 Fig — (TIF) [file ppat.1009795.s003.tif]

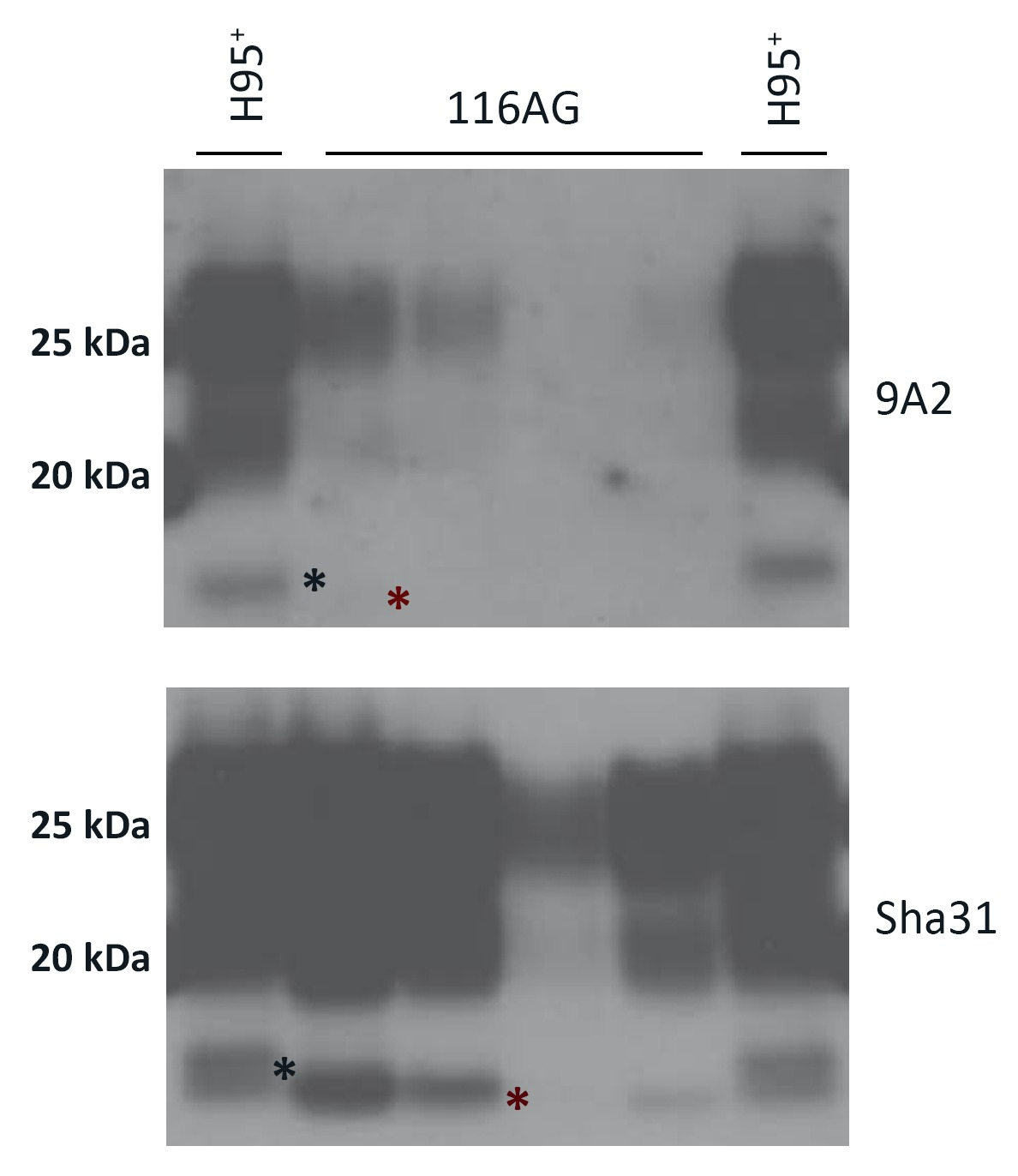

Supplement: S4 Fig — Western blot of brain homogenates from mice inoculated with, H95+ or 116AG prions after PK digestion. The same samples have been loaded on the same gel twice, then cut into two to be probed with different mAbs. PrPres was detected using the N-terminal anti-prion monoclonal antibody 9A2 (upper panel) and a central-region anti-prion monoclonal antibody sha31 (lower panel). The asterisks show the migration profile, slow (black asterisk) and rapid (red asterisk), of the non-glycosylated bands. (TIF) [file ppat.1009795.s004.tif]

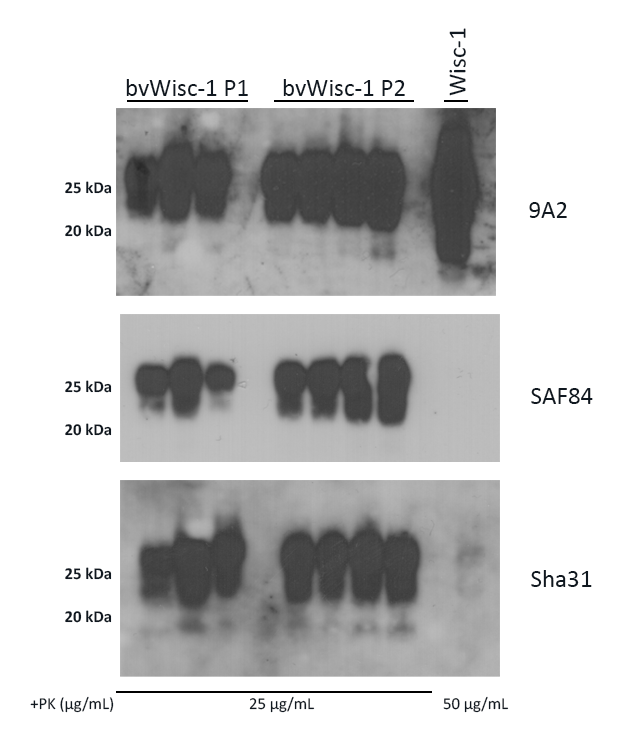

Supplement: S5 Fig — Inoculations of bank voles were performed through the intracerebral route using 20 ul of 1% (w/v) brain homogenate. Western blot analyses were performed using mAbs (A) 9A2 (aa 102 to 104), (B) SAF84 (aa 167 to 173), and (C) Sha31 (aa 143 to 153) depicting PrPres in brain homogenates of bank voles inoculated with Wisc-1 upon first passage (lanes 1–3), second passage (lanes 5–8) and Wisc-1 original isolate (lane 10). Results with different antibodies were obtained from individual western blots of the same samples. (TIF) [file ppat.1009795.s005.tif]

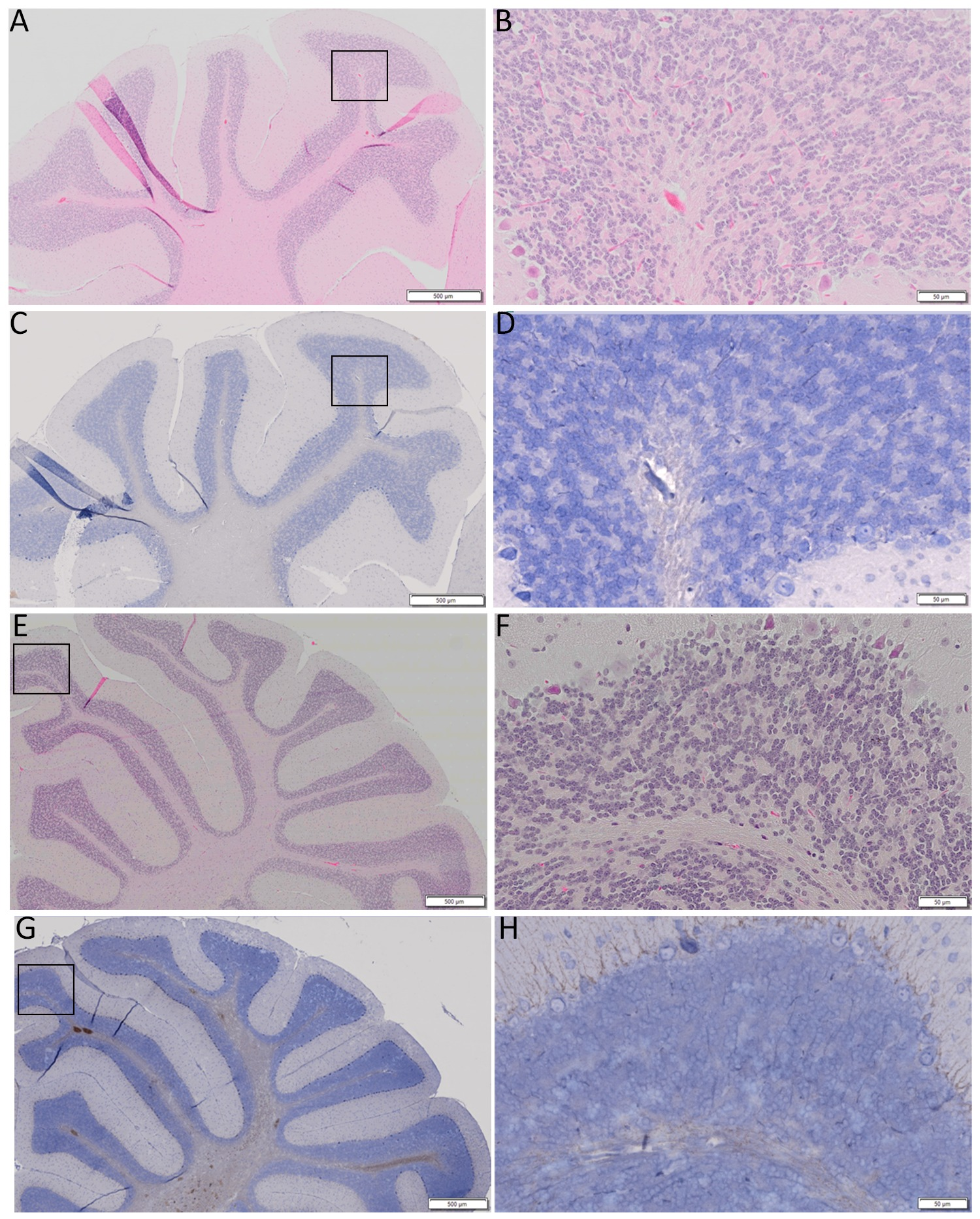

Supplement: S6 Fig — H&E staining (A-B and E-F) and immunostaining of PrP (C-D and G-H) of brain sections of tg1536+/+ mice inoculated with Wisc-1 (A-D) or 116AG-short (E-H) confirms the absence of unicentric plaques in the cerebellum of these animals. Scale bars indicate 50 μm (B, D, F and H) as high magnification and 500 μm (A, C, E and F). (TIF) [file ppat.1009795.s006.tif]
